# Evaluation of the Antimicrobial Activity of a 3% Chlorhexidine/Ophytrium Mousse and Shampoo in 18 Healthy Dogs

**DOI:** 10.1111/vde.70057

**Published:** 2026-02-27

**Authors:** Matt McHale, Sonya Bettenay, Gavin K. Paterson, Tim Nuttall, Victoria Robinson

**Affiliations:** ^1^ Royal (Dick) School of Veterinary Studies The University of Edinburgh Edinburgh UK; ^2^ Tierdermatologie Consultations Oberhaching Germany

**Keywords:** chlorhexidine, compliance, dysbiosis, hair, mousse, shampoo

## Abstract

**Background:**

Topical antimicrobial therapy is vital for managing pyoderma yet poor compliance can affect treatment outcomes.

**Hypothesis/Objective:**

To evaluate the antimicrobial activity of a 3% chlorhexidine/ophytrium shampoo at different contact times and the residual activity of a 3% chlorhexidine/ophytrium mousse against meticillin‐susceptible and ‐resistant *Staphylococcus pseudintermedius* (MSSP and MRSP), *Escherichia coli* (EC), ESBL‐producing *E. coli* (ESBL), *Pseudomonas aeruginosa* (PA), and *Malassezia pachydermatis* (MP) in vitro.

**Animals:**

18 privately owned dogs.

**Materials and Methods:**

Dogs were treated once with the shampoo for 3, 5 and 10 min, with hair samples taken after rinsing. The mousse was applied once. Hair samples were collected at 30 min (Day [D]0) and at D2, D4, D7 and D14 post‐treatment. Hair bundles were plated on Mueller–Hinton (MH) agar plates pre‐inoculated with MSSP, MRSP, EC, ESBL and PA, and on supplemented MH plates pre‐inoculated with MP. Zones of inhibition (ZIs) were measured after 24 h (bacteria) and 48 h (MP) incubation.

**Results:**

Shampoo—there were no significant differences in antimicrobial activity between the contact times for any of the isolates. Mousse—for all the isolates, there was significant growth inhibition compared to the negative controls on D0, D2 and D4.

**Conclusions and Clinical Relevance:**

Hairs exposed to the shampoo and mousse inhibited growth of all the isolates in vitro. A 3 min contact time was noninferior to 10 min for the shampoo. The mousse showed residual activity for ≥ 4 days for MSSP, MRSP, EC, PA and MP.

## Graphical abstract

Background: Topical antimicrobial therapy is vital for managing pyoderma yet poor compliance can affect treatment outcomes.Hypothesis/objective: To evaluate the antimicrobial activity of a 3% chlorhexidine/ophytrium shampoo at different contact times and the residual activity of a 3% chlorhexidine/ophytrium mousse against meticillin‐susceptible and ‐resistant *Staphylococcus pseudintermedius* (MSSP and MRSP), *Escherichia coli* [EC], ESBL‐producing *E. coli* (ESBL), *Pseudomonas aeruginosa* (PA) and *Malassezia pachydermatis* (MP) in vitro. Conclusions and Clinical Relevance: Hairs exposed to the shampoo and mousse inhibited growth of all the isolates in vitro. A 3 min contact time was noninferior to 10 min for the shampoo. The mousse showed residual activity for ≥4 days for MSSP, MRSP, EC, PA and MP.

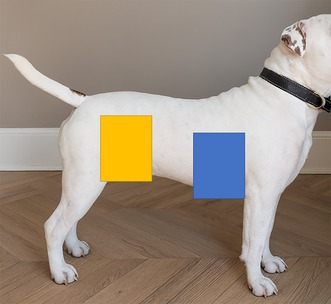

## 1 Introduction

Microbial dysbiosis and infection is common in dogs with skin disorders [1, 2, 3]. With the emergence of meticillin and multidrug resistance (MDR) [3, 4] there is a need to reduce systemic antimicrobial use. Antimicrobial resistance (AMR) is not limited to bacteria and includes fungi of significance in human and animal health [5]. MDR *Candida auris* has been isolated from the oral cavity and from the skin of dogs [6, 7], yet its relevance to canine health is unclear. However, emerging azole resistance in *Malassezia pachydermatis* [8] has been associated with treatment failure [9].

Topical antimicrobials should be used as first‐line treatment for surface and superficial pyoderma [3, 10], and as adjunctive therapy for deep pyoderma [3, 11]. Multiple formulations are available, such as shampoos, mousses, pads, sprays, and spot‐ons. Their ease of use and frequency of application are variable, with some protocols suiting animal and owner needs more than others [12]. However, most topical antimicrobials are not licensed medicines and therefore rigorous data on efficacy and the timing of treatment are not always available. It is important that clinicians have access to such information to select and use topical antimicrobials effectively.

Contact times for antimicrobial shampoos are often cited as 10 min, yet to the best of the authors' knowledge, this recommendation is arbitrary. A lack of sufficient time is frequently identified by owners as a major barrier to using topical therapies [13]. Loss of time, whether through treatment application or repeated veterinary visits, impacts the quality‐of‐life (QoL) of owners of dogs with atopic dermatitis (AD) [14, 15, 16]. On the one hand, reducing bathing contact time and frequency may facilitate treatment and improve clinical outcomes [17]. On the other, reduced efficacy could delay resolution and affect patient outcomes. Topical antimicrobial therapy should therefore be optimised to improve clinical outcomes and antimicrobial stewardship.

The objective of this study was to use a modified version of an established in vitro protocol [10, 18, 19, 20, 21] to evaluate the antimicrobial activity of a 3% chlorhexidine/ophytrium shampoo at different contact times, and the residual activity of a 3% chlorhexidine/ophytrium mousse against meticillin‐susceptible *Staphylococcus pseudintermedius* (MSSP), meticillin‐resistant *S. pseudintermedius* (MRSP), *Escherichia coli* (EC), ESBL‐producing *E. coli* (ESBL), *Pseudomonas aeruginosa* (PA) and *Malassezia pachydermatis* (MP) in vitro. A secondary aim was to assess differences in antimicrobial activity of the products between MSSP versus MRSP and EC versus ESBL.

Our hypotheses were:

1. A 3 min contact time is noninferior to a 10 min contact time for the 3% chlorhexidine/ophytrium shampoo.
2. There is residual antimicrobial activity of the 3% chlorhexidine/ophytrium mousse for ≥ 4 days (i.e., equating to twice‐weekly application).
3. There is no significant difference in antimicrobial efficacy of the 3% chlorhexidine/ophytrium shampoo and mousse for MSSP versus MRSP and EC versus ESBL.

## 2 Materials and Methods

### 2.1 Ethics

This prospective study was approved by the Royal (Dick) School of Veterinary Studies Veterinary Ethical Review Committee (reference 1.24a). Informed written consent was obtained from all owners before inclusion.

Nineteen privately owned, healthy dogs were included. Exclusion criteria included: treatment with oral antibiotics, antifungals or glucocorticoids within 30 days, long‐acting glucocorticoid medication within 42 days, and topical antibiotics, antifungals or antiseptics within 21 days of inclusion; any history of nonparasitic pruritus, skin lesions and/or ectoparasite infestation within 90 days of inclusion; and existing cutaneous neoplasia, endocrinopathy or autoimmune dermatoses.

Nine dogs were included in the mousse study only, nine dogs in the shampoo study only, and one dog in both parts of the study. Allocation to each group was dependent on owner preference and availability, as well as patient temperament for bathing or mousse application.

### 2.2 Study Protocol—Shampoo

**FIGURE 1:**
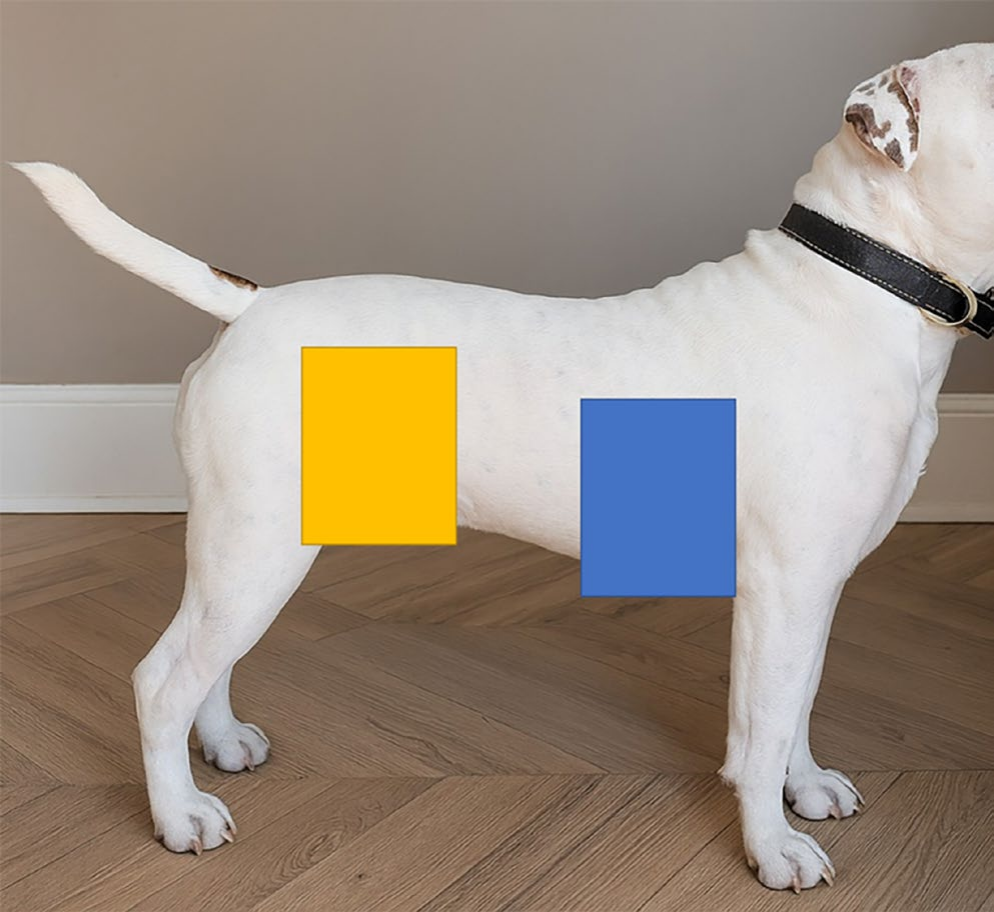
Sample areas used to evaluate the antimicrobial activities of a 3% chlorhexidine/ophytrium shampoo and mousse. The blue area on the right and the left side of the thorax was used for the mousse study. The blue and yellow areas were used on the right and left sides for the shampoo areas, with contact times rotated to minimise confounding factors.

Each dog was bathed by one of the primary investigators (MM or SB) using a set protocol. Each dog was rinsed with warm water from the top of the head to the tail, sparing the face. Once the coat was wet, a control sample was taken from one of the four test quadrants (Figure 1). Samples were taken with blunt/blunt scissors, cut as close to the skin as safely possible. A different pair of scissors was used for each dog, and the scissors were cleaned with a 70% alcohol swab (Alcotip, 365 Healthcare, UK) and air‐dried to allow evaporation of the alcohol before sampling. A 3% chlorhexidine/ophytrium shampoo (Douxo S3 Pyo; Ceva) was then applied to the whole coat at a dose of 1 pump per 2 kg body mass, as per the manufacturer's guidelines for short‐/medium‐coated dogs that weighed > 6 kg. The dose was doubled for dogs with a long or dense coat.

A timer was set for 3, 5, and 10 min. The shampoo was rinsed thoroughly from the site to be sampled at each time point and hair clipped with cleaned scissors as above. The hair samples were collected into separate, labelled, plastic bags wearing gloves, which were changed between each sample.

The sites used for each time point were rotated between dogs to minimise possible confounding factors including hair density/length and sebum differences. Four sites were used for each dog, including the left and right axillae, and left and right flanks (Figure 1). Negative control samples without treatment also were collected before shampoo application.

### 2.3 Study Protocol—Mousse

The protocol was modified from previous studies [10, 18, 19, 20, 21]. On D0, a 3% chlorhexidine/ophytrium mousse (Douxo S3 Pyo; Ceva) was applied as per the manufacturer's guidelines. The product was massaged into the skin by hand without rinsing, using gloves to avoid any contamination or residual chemicals from hands. The dose was one pump per 2 kg for dogs, as per the manufacturer's guidelines for short‐/medium‐coated dogs. The dose was doubled for dogs with a long or dense coat.

Hair samples were taken on D0 (within 1 h of mousse application), D2, D4, D7, and D14 using blunt/blunt scissors as above. Samples were taken from a set area on either the left or right side of the thorax of each dog (Figure 1). Negative control samples without treatment also were collected before mousse application on D0.

### 2.4 Laboratory Protocol—Mousse and Shampoo

Isolates of MSSP (DSM 21284), MRSP (in‐house canine pyoderma isolate 6109), EC (NCTC 10418), ESBL (NCTC 13351), PA (ATCC 27853) and MP (NCPF 3667) were recovered from cryovials and inoculated onto Columbia blood agar with 5% (v:v) defibrinated horse blood (E&O labs, Bonnyrigg, UK) for the bacterial isolates and Mueller–Hinton (MH) agar with 2% glucose and methylene blue for MP (E&O labs). The plates were incubated at 37°C overnight. Colonies were re‐suspended in sterile saline to a density equivalent to a 0.5 McFarland turbidity standard for the bacteria and a 2.0 McFarland turbidity standard for MP.

**FIGURE 2:**
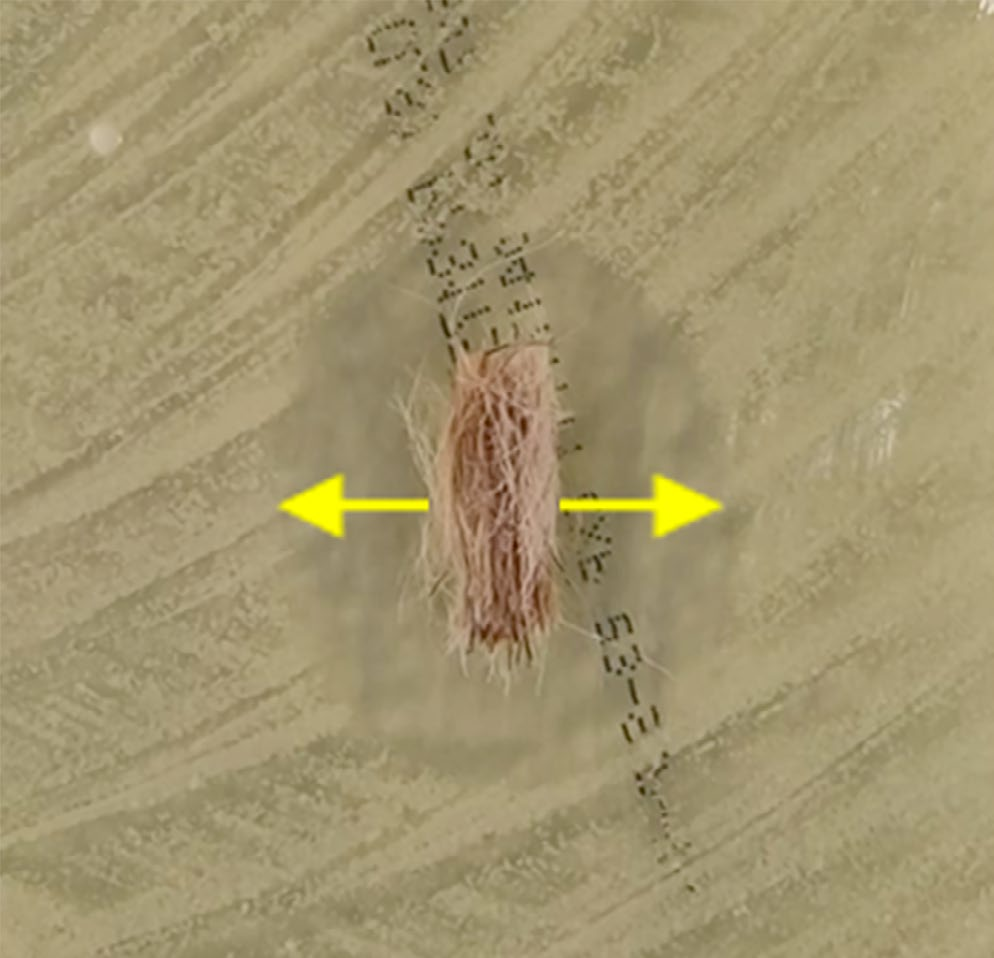
Plated bundle of hair on agar inoculated with bacteria. The yellow arrows display the measurements from the hair to the edge of the zone of inhibition.

Test plates were streaked with the inocula. A new sterile cotton‐tipped swab was used for each plate, and plates streaked with different isolates were kept separate. The entire surface of the plates was streaked three times, with 120° rotations made between each streak. Hair bundles were placed onto the centre of each plate immediately after inoculation. The hair had been trimmed before plating to achieve consistent hair bundles weighing approximately 0.01 g, which were cultured in triplicate with one hair bundle per test plate. All hair samples were plated within 8 h of sampling. All plates were incubated at 37°C. Bacterial plates were incubated for 24 h and MP plates for 48 h before measuring the zones of inhibition (ZI). The distance from the hair bundle to the edge of the ZI was measured at the mid‐hair shaft on each side (Figure 2). Both values were added together and divided by two to get the mean ZI (in mm).

### 2.5 Statistical Methods

Statistical analyses were performed using prism 10.4.0 for Windows (GraphPad). All six values (two sides, performed in triplicate) were averaged for each sample before analysis. Shapiro–Wilk testing was performed on all data sets to determine normality, with *p*‐values > 0.05 considered as normally distributed. For parametric data, Student's *t*‐tests or ANOVA testing was performed. For nonparametric data, Kruskal–Wallis or Wilcoxon–Mann–Whitney *U*‐testing was performed. Tukey's honestly significant difference or Dunn's multiple comparison testing was performed on all pairwise comparisons. Wilcoxon signed rank testing was performed to compare the test samples to the negative controls. Significance was set at 0.05.

For the mousse protocol, each time point was tested against the negative control (i.e., samples before mousse application) for all isolates to demonstrate antimicrobial activity and identify the optimal dosing frequency. Further analysis was performed to assess differences (i.e., MSSP, MRSP, EC, ESBL, PA and MP) at D0 (following mousse application) and the successive time points (D2, D4, D7 and D14) to assess the rate of decline of antimicrobial activity.

For the shampoo protocol, initial testing was performed to assess variance between the three time points (3, 5 and 10 min contact time). Direct comparison was then performed on the datasets for 3 and 10 min contact times.

Further testing compared the results for MSSP versus MRSP and EC versus ESBL at all five time points for the mousse and between all three contact times for the shampoo.

## 3 Results

Eighteen dogs completed the study, with one dog (Dog 9) removed retrospectively from the mousse study, owing to a swimming incident on D1. The study population of the mousse group included three short‐haired pure‐bred dogs, five long‐haired pure‐bred dogs, and one short‐haired mixed‐breed dog. The study population of the shampoo group included five long‐haired pure‐bred dogs, two short‐haired pure‐bred dogs, and three short‐haired mixed‐breed dogs. No adverse reactions to the topical interventions were seen. There was no inhibition of microbial growth on any negative control samples.

### 3.1 Shampoo—Contact Time

There was no significant difference between the three contact times for MSSP, MRSP, EC, ESBL, PA or MP (*p* = 0.3, *p* = 0.1, *p* = 0.2, *p* = 0.4, *p* = 0.5 and *p* = 0.5, respectively). To further test the hypothesis, the 3 and 10 min contact times were directly analysed, with no significant differences for any of the tested microbes (*p* = 0.2, *p* = 0.5, *p* = 0.5, *p* = 0.6, *p* = 0.4 and *p* = 0.2 for MSSP, MRSP, EC, ESBL, PA and MP, respectively; Table 1).

**TABLE 1:** Shampoo: Mean zones of inhibition ± standard deviation (minimum, maximum).

Shampoo	Zones of inhibition (mm)		
	3 min	5 min	10 min
MSSP	1.84 ± 0.64 (0.92, 2.83)	1.79 ± 0.58 (0.92, 3.08)	2.16 ± 0.53 (1.42, 2.83)
MRSP	1.79 ± 0.57 (1, 2.75)	1.35 ± 0.25 (1, 1.75)	1.63 ± 0.5 (0.83, 2.33)
EC	1.8 ± 0.75 (0.92, 3.42)	1.5 ± 0.49 (0.75, 2.58)	2.04 ± 0.76 (0.92, 3.08)
ESBL	1.07 ± 0.85 (0, 3.08)	0.98 ± 0.77 (0.17, 3)	1.24 ± 0.72 (0.17, 2.75)
PA	1.81 ± 1.41 (0.86, 5.67)	1.9 ± 0.87 (1.08, 3.92)	2.18 ± 1.44 (0.42, 5.67)
MP	1.37 ± 0.79 (0.42, 3.17)	1.59 ± 0.68 (0.58, 2.67)	1.93 ± 1.36 (0, 5.33)

Abbreviations: EC, *Escherichia coli* ; ESBL, ESBL‐producing *E. coli* ; MP, *Malassezia pachydermatis*; MRSP, meticillin‐resistant *S. pseudintermedius* ; MSSP, meticillin‐susceptible *Staphylococcus pseudintermedius* ; PA, *Pseudomonas aeruginosa.*

### 3.2 Shampoo—Isolates Comparison

The 3 and 10 min contact time points were directly compared between MSSP and MRSP (*p* = 1.0, *p* = 0.2, respectively), and EC and ESBL (*p* = 0.2, *p* = 0.2, respectively; Table 1).

### 3.3 Mousse—Residual Activity

For all the isolates, there was a significant difference from the negative control on D0, D2, and D4 (*p* > 0.05; Table 2). There was no significant difference from the negative control on D7 and D14 for ESBL, PA, and MP.

**TABLE 2:** Mousse: Mean zones of inhibition ± standard deviation (minimum, maximum).

Mousse	Zones of inhibition (mm) by time point (Day)				
	D0 (1 h)	D2	D4	D7	D14
MSSP	5.57 ± 1.65 (3.08, 7.75)*	4.38 ± 1.58 (1.5, 6.58)*	2.94 ± 1.64 (1, 5.33)*	1.35 ± 1.53 (0, 4.83)*	0.65 ± 0.64 (0, 1.67)*
MRSP	5.85 ± 1.65 (3.5, 8.67)*	4.13 ± 1.71 (1.25, 6.17)*	2.55 ± 1.45 (0.92, 4.92)*	1.25 ± 1.5 (0, 4.83)*	0.5 ± 0.59 (0, 1.58)*
EC	4.63 ± 1.8 (1.67, 7.42)*	3.91 ± 1.27 (2.08, 6)*	2.24 ± 1.3 (0.75, 4.58)*	1.11 ± 1.68 (0, 5.08)*	0.27 ± 0.34 (0, 0.92)
ESBL	5.07 ± 2.11 (2.17, 7.5)*	2.44 ± 1.41 (0, 4.33)*	1.85 ± 1.31 (0, 3.58)*	0.56 ± 1 (0, 3)	0.14 ± 0.19 (0, 0.5)
PA	2.45 ± 0.91 (1.08, 3.58)*	2.81 ± 0.97 (0.67, 4.17)*	1.68 ± 0.9 (0.5, 2.75)*	0.55 ± 0.88 (0, 2.42)	0.26 ± 0.32 (0, 0.75)
MP	4.05 ± 3.15 (0, 8.42)*	3.37 ± 1.65 (1.42, 6.08)*	1.7 ± 0.94 (0.75, 3.75)*	0.31 ± 0.86 (0, 2.58)	0.04 ± 0.11 (0, 0.33)

Abbreviations: EC, *Escherichia coli* ; ESBL, ESBL‐producing *E. coli* ; MP, *Malassezia pachydermatis*; MRSP, meticillin‐resistant *S. pseudintermedius* ; MSSP, meticillin‐susceptible *Staphylococcus pseudintermedius* ; PA, *Pseudomonas aeruginosa* .

* Significant difference (*p* < 0.05) from the negative control samples.

There were no significant differences in residual antimicrobial activity between D0 and D2 for MSSP (*p* = 0.4), MRSP (*p* = 0.1), and EC (*p* = 0.8), and between D0 and D2 or D4 for PA (*p* = 0.9, *p* = 0.3, respectively) and MP (*p* = 0.8, *p* = 0.2, respectively), suggesting the optimal dosing frequency of the mousse for MSSP, MRSP, EC, PA, and MP isolates would be approximately every 2–4 days. There was a significant difference between D0 and all other time points for ESBL; the more rapid decline in antimicrobial efficacy suggests that more frequent application may be necessary for this bacterial isolate.

### 3.4 Mousse—Isolates Comparison

There were no significant differences between MSSP and MRSP (*p* = 1.0) or EC and ESBL at any time point (*p* = 0.28, *p* = 1.0, *p* = 1.0, *p* = 1.0 for D2, D4, D7 and D14, respectively; Table 2).

## 4 Discussion

The current study demonstrated that hair samples exposed to a 3% chlorhexidine/ophytrium mousse and shampoo show in vitro antimicrobial activity against isolates of MSSP, MRSP, EC, ESBL, PA, and MP. Moreover, there was noninferior in vivo/in vitro activity of the shampoo with a 3 min compared to a 10 min contact time. The results also suggest that hairs treated with the mousse show in vitro antimicrobial activity for at least 2–4 days. The shorter bathing time and twice‐weekly mousse application may therefore simplify treatment and increase compliance. By contrast, the ESBL isolate demonstrated significant loss of antimicrobial efficacy as soon as 48 h after application of the mousse, so every other day treatment may be considered dependent on cytological results and/or bacterial culture and antimicrobial susceptibility testing (AST). However, these protocols should be tested in treatment trials as clinical efficacy cannot be assumed from the in vitro activity, and the results of this study should not be used to assume in vivo efficacy. In particular, evaluating hair bundles instead of the surface of the skin may lead to overestimation of actual antimicrobial effects [20].

These results confirm those of previous in vitro studies using hair bundles to evaluate the efficacy and/or residual activity of topical chlorhexidine products against MSSP [10, 18, 19, 20, 21]. Chlorhexidine shampoos, mousses, pads/wipes, and sprays have in vivo and in vitro efficacy against a number of micro‐organisms [10, 18, 19, 20, 21, 22, 23, 24, 25]. One study showed less inhibition of growth with a shampoo than with a mousse [21]. This difference may be a result of the level of diffusion of the chlorhexidine across the agar plates between the vehicles [19, 20]. However, this also may be a consequence of the treatment protocol, as the shampoo was rinsed thoroughly before sampling, while the mousse was applied and remained. Direct comparison between the shampoo and mousse products was therefore not considered.

As per Wu et al. [20], the size of ZIs can be used as a marker of distribution of the mousse product. The previous study investigated hair bundles and skin‐surface swabs, with ZIs from skin swabs significantly smaller from long‐haired dogs compared to short haired dogs [20], suggesting that hair length may be a barrier to product distribution to the skin surface. Wu et al. [20] used one pump of mousse per 5 × 5 cm^2^ area independent of hair length, while the current study used double the number of pumps for long‐haired dogs, compared to short‐haired dogs.

This study did not aim to standardise the precise volume of shampoo or mousse applied to each patient to mimic the clinical scenario. A previous study postulated differences in volume per pump, surface area per pump, and viscosity of the mousse as possible causes of differences in distribution [20]. As the same shampoo and mousse were used for each patient, the differences in viscosity and surface area per pump are assumed to be minimal; however, it is possible that there was some discrepancy based on user, storage scenario, and interbatch differences between bottles. Two of the authors (MM or SB) were involved in application of any product, and so user differences were minimised, where possible.

The activity of the ophytrium in vitro is unclear. In experimental canine skin models, it limited adhesion of *Staphylococcus pseudintermedius* to keratinocytes, reduced biofilm formation [26], promoted normal skin morphology, reduced trans epidermal water loss, and reduced secretion of pro‐inflammatory cytokines (interleukin [IL]‐8, IL‐13 and thymic stromal lymphopoietin [TSLP]) [27]. It is possible that ophytrium influences antimicrobial activity efficacy in vivo. The role of other inactive ingredients contained within the shampoo or mousse remains unknown; however, these may play a role in vitro and in vivo [20].

The tested isolates were all clinically relevant to canine pyoderma and microbial dysbiosis. The canine microbiome is diverse, and altered skin barrier function and inflammation can skew populations resulting in dysbiosis [2, 28]. *S. pseudintermedius* and *M. pachydermatis* are the most common pathogens associated with dysbiosis in canine AD [29, 30]. However, other pathogens identified in skin infections include *E. coli* [31, 32, 33] and *P. aeruginosa* [32, 33, 34]. AMR isolates also were included in this study, yet no significant differences in activity against MSSP versus MRSP and EC versus ESBL were seen. However, when comparing D0 data against subsequent days, antimicrobial activity of the mousse declined more rapidly for the ESBL isolate, suggesting that these AMR genes may affect the residual antimicrobial activity of the products. Clinical resistance to chlorhexidine has been observed [35, 36] yet its association with antibiotic resistance is unclear. Some studies have found higher minimum inhibitory concentrations (MICs) for AMR bacterial isolates compared to sensitive isolates of the same species [37, 38, 39, 40]. Chlorhexidine use has been hypothesised to lead to cross‐resistance or co‐resistance to antibiotics [40, 41, 42, 43]. Possible mechanisms include acquisition of *qacA/B*‐encoding plasmids [40], decreased cell envelope permeability [44] and increased efflux pump activity [45]. However, AMR does not necessarily correlate with higher MICs to chlorhexidine, as for *qacA/B* in *S. pseudintermedius* [46].

The ZIs for PA were smaller than those for MSSP and MRSP after application of the mousse. MICs for chlorhexidine are lower for Gram‐positive than Gram‐negative bacteria [47, 48], owing to increased permeability of Gram‐positive bacterial cell walls [49]. However, there was no significant difference between the staphylococcal isolates and the *E. coli* isolates, which may suggest an alternative mechanism for reduced susceptibility of this PA isolate to chlorhexidine when compared to MSSP and MRSP. The use of only a single isolate of each tested microbe limited this study, as there may be differences between isolates within species. Further studies should be performed to investigate repeatable patterns between and within microbial species.

### 4.1 Limitations

The study had a number of limitations. The relatively low sample size of this pilot study reduced the statistical power to detect significant differences between the time points for both the mousse and shampoo protocols. The study design did not test residual activity of the shampoo, although this has been demonstrated previously for ≥ 7 days [10]. Unlike previous studies, pre‐treatment bathing with a nonmedicated shampoo was not performed to mimic a ‘real life’ scenario with minimal other interventions. The hair bundle size used was 0.01 g, which is the same as one of the previous studies [21] yet only half that used in the other studies [10, 18, 19, 20]. This study used a 0.5 McFarland inoculum as with Wu et al. [20], while the others used a 0.3 McFarland inoculum [10, 18, 19, 21]. Additionally Taketa et al. [21] used the same 3% chlorhexidine and 0.5% ophytrium mousse, while the other studies used 3% chlorhexidine containing climbazole and/or phytosphingosine salicyloyl [18, 19, 20]. This limits the relevance of comparison between the studies.

The hair bundles were trimmed and weighed to reduce the variance between samples, yet it was impossible to ensure that all samples were identical. It is possible that distal hair shafts had a higher exposure to the products, as a previous study showed smaller ZIs for long‐haired than short‐haired dogs [20]. However, it is also possible that longer primary hairs protect the undercoat and allow greater product retention nearer the skin. In the current study, differences between short‐ and long‐haired dogs were not analysed owing to the low numbers of each coat type in each group. Wu et al. proposed that these protocols [10, 18, 19, 20, 21] may not be appropriate for evaluating the effects of topicals on the skin surface of long‐haired dogs, and that skin‐surface swabs may be more appropriate for investigation of product distribution in long‐haired dogs [20].

## 5 Conclusions

This study supports the use of these topical chlorhexidine‐containing shampoos and mousses for micro‐organisms (MSSP, MRSP, EC, ESBL, PA and MP) involved in cutaneous dysbiosis or pyoderma. There was no difference in the activity against AMR and sensitive staphylococci and *E. coli* . The results suggest that a 3 min contact time for the shampoo and twice‐weekly application of the mousse may be as effective as more intensive regimens. However, clinical trials are needed to test the in vivo efficacy of the proposed 3 min shampoo contact time and twice‐weekly mousse application, as inhibition of microbial growth with treated hair bundles in vitro, is not equivalent to managing pyoderma or *Malassezia* dermatitis in vivo. ESBL‐producing *E. coli* may require more frequent application of chlorhexidine‐based products, and further study is required to assess this finding.

It is important to note that the ZIs in this study do not imply sensitivity or resistance of the tested microbial isolates to the products. First, ASTs cannot be used with topical therapies as the break‐points to determine susceptibility or resistance are based on tissue concentrations following systemic administration [50]. Additionally, the ability of the individual components of mousses and shampoos to diffuse into agar is unknown, and so the ZIs may under‐ or overestimate the activity of chlorhexidine and/ophytrium on canine skin [19, 20].

This pilot study will be used as justification to test the proposed altered protocol for in vivo management of pyoderma in atopic dogs, with the standard 3% chlorhexidine/ophytrium shampoo and mousse protocol used as a control.

## Author Contributions

**Sonya Bettenay:** data curation, investigation, methodology, writing – review and editing. **Victoria Robinson:** conceptualization, supervision, funding acquisition, methodology, writing – review and editing. **Gavin K. Paterson:** methodology, resources, validation, writing – review and editing. **Tim Nuttall:** conceptualization, formal analysis, methodology, writing – review and editing. **Matt McHale:** conceptualization, methodology, investigation, funding acquisition, writing – original draft, visualization, data curation, formal analysis, writing – review and editing.

## Funding

This study was funded by the European Society of Veterinary Dermatology (ESVD) minor grant.

## Conflicts of Interest

Ceva has provided material support for the dermatology clinic at the Royal (Dick) School of Veterinary Studies within the last 5 years. However, Ceva was not involved in the planning, financing, analysis or writing of this study and manuscript. T.N. has received lecture fees and other funding from Ceva in the last 5 years. V.R. has received travel support from Ceva in the last 5 years. None of the other authors have any conflicts of interest to declare.

## Supporting information


**Data S1:** Raw data from the sampling of all 10 dogs involved in the mousse portion of the study.

**Data S2:** Raw data from the sampling of all 10 dogs involved in the shampoo portion of the study.

## Data Availability

The data that supports the findings of this study are available in Supporting Information of this article.
